# Supplementary material for: Glia control experience-dependent plasticity in an olfactory critical period
Source: bioRxiv. 2024 Jul 5:2024.07.05.602232. Preprint. [Version 1] doi: 10.1101/2024.07.05.602232 (PMC11245089; doi:10.1101/2024.07.05.602232)
Supplement: Supplementary file 1 [file NIHPP2024.07.05.602232V1-supplement-1.pdf]

Supplemental Table 1. Genotypes used in experiments.

Figure	Genotype
Figure 1D–J	*w; or42a-mcd8::GFP/+; repo-Gal4/+*
Figure 2D–I	*w; or42a-mcd8::GFP/+; repo-Gal4/+*
Figure 2K	*w; or22a-Gal4/+; UAS-mcd8::GFP/+*
Figure 3	*w; or42a-mcd8::GFP/+; repo-Gal4/+*
Figure 4B	*w; ; GMR56F03-Gal4/UAS-mcd8::GFP*<br>*w; ; GMR86E01-Gal4/UAS-mcd8::GFP*
Figure 4C–D	*w; or42a-mcd8::GFP/+; repo-Gal4/+*<br>*w; or42a-mcd8::GFP/UAS-draper-I RNAi; repo-Gal4/+*<br>*w; or42a-mcd8::GFP/+; repo-Gal4/UAS-draper RNAi*
Figure 5A–B	*w; or42a-mcd8::GFP/UAS-mcd8::RFP; alrm-Gal4/UAS-luciferase*<br>*w; or42a-mcd8::GFP/UAS-mcd8::RFP; alrm-Gal4/UAS-draper RNAi*<br>*w; or42a-mcd8::GFP/UAS-draper-I RNAi; alrm-Gal4/UAS-mcd8::RFP*
Figure 5C–D	*w; or42a-mcd8::GFP/UAS-mcd8::RFP; GM456F03-Gal4/UAS-luciferase*<br>*w; or42a-mcd8::GFP/UAS-mcd8::RFP; GM456F03-Gal4/UAS-draper RNAi*<br>*w; or42a-mcd8::GFP/UAS-draper-I RNAi; GM456F03-Gal4/UAS-mcd8::RFP*
Figure 6	*w; or42a-mcd8::GFP/UAS-mcd8::RFP; GM456F03-Gal4/UAS-luciferase*<br>*w; or42a-mcd8::GFP/UAS-mcd8::RFP; GM456F03-Gal4/UAS-draper RNAi*<br>*w; or42a-mcd8::GFP/UAS-draper-I RNAi; GM456F03-Gal4/UAS-mcd8::RFP*
Figure 7A–C	*w; UAS-mcd8::RFP/+; GM456F03-Gal4/Mi{MIC}draper::EGFP-FlAsH-StrepII-TEV-3xFlag*
Figure 7D–F	*w; GMR56F03-LexA/or42a-Gal4; UAS-pHluorin::tdTomato/+*<br>*w; GMR56F03-LexA/or42a-Gal4; UAS-pHluorin::tdTomato/LexAop-draper RNAi #1*<br>*w; GMR56F03-LexA/LexAop-draper RNAi #2; UAS-pHluorin::tdTomato/or42a-Gal4*
